# Melatonin in the Regulation of Liver Steatosis following Prenatal Glucocorticoid Exposure

**DOI:** 10.1155/2014/942172

**Published:** 2014-04-13

**Authors:** Mao-Meng Tiao, Li-Tung Huang, Chih-Jen Chen, Jiunn-Ming Sheen, You-Lin Tain, Chih-Cheng Chen, Ho-Chang Kuo, Ying-Hsien Huang, Kuo-Shu Tang, En-Wei Chu, Hong-Ren Yu

**Affiliations:** Department of Pediatrics, Kaohsiung Chang Gung Memorial Hospital and Chang Gung University College of Medicine, Kaohsiung 833, Taiwan

## Abstract

Nonalcoholic fatty liver disease patients are characterized by hepatic steatosis. Prenatal glucocorticoid overexposure can result in steatosis. In this study, we aimed to determine the mechanism and cellular apoptosis of prenatal glucocorticoid overexposure in rats and whether melatonin can rescue the prenatal glucocorticoid-induced steatosis and apoptosis in neonatal rats. Pregnant Sprague-Dawley rats at gestational days 14 to 21 were administered dexamethasone. Acute effects of prenatal programming liver were assessed at postnatal day 7. The expression of proteins involved in the apoptotic and methylation pathways was analyzed by RT-PCR and Western blotting. Apoptosis and steatosis were examined by histology staining. The liver steatosis and apoptosis were increased in prenatal glucocorticoid group more than in control group and decreased in melatonin group. The expression of leptin decreased in prenatal glucocorticoid and increased in melatonin group by liver RT-PCR and Western blot study. Caspase 3, TNF-**α** proteins expression, and TUNEL stains increased in prenatal glucocorticoid compared with control and decreased in melatonin group. The liver histone deacetylase, DNA methyltransferase activity, and DNA methylation were increased in prenatal glucocorticoid and decreased in melatonin group. The present study showed that the prenatal glucocorticoid induced programming liver steatosis at day 7 after delivery, possibly via altered leptin expression. Melatonin can reverse the methylation of leptin and decreased liver steatosis.

## 1. Introduction

Approximately 7% of pregnant women are at risk of preterm delivery and these women are routinely treated with synthetic glucocorticoids to improve neonatal outcome [1]. Factors such as prenatal exposure to glucocorticoids can all have negative health consequences for the offspring that persist into adulthood [2, 3]. Prenatal glucocorticoids overexposure in rats increases susceptibility to fatty liver disease. This may be due to depot-specific-programmed alterations in fat metabolism [4, 5]. Regulatory regions of the human genome can be modified through epigenetic processes during prenatal life to make an individual more likely to suffer from diseases when they reach adulthood [6]. Thus, it is important to plan a strategy to rescue the liver steatosis.

The adipocyte hormone leptin is a critical modulator of both acute appetitive state and long-term metabolic health. Leptin was initially described as an adipostatic signal controlling food intake and energy expenditure [7] and body weight regulation [8]. Circulating leptin levels correlate with adiposity, rising with fat accumulation and falling with its depletion [9].

Melatonin is the major product of the pineal gland. Moreover, this hormone is an antioxidant and has broad effects on inflammatory cells [10, 11]. It is reported that melatonin reduced serum total cholesterol and triglycerides [12] and may have therapeutic effect in fatty liver disease [12].

The implication of different prenatal damage opens a door to manipulate the liver disease. However, the effects of manipulation of molecules on prenatal glucocorticoid exposure are unknown. We thus hypothesized that the prenatal glucocorticoid exposure on the programming effect and melatonin may determine pathogenesis of liver steatosis.

## 2. Materials and Methods

### 2.1. Animals

Sprague-Dawley (SD) rats were housed in the animal care facility in Chang Gung Memorial Hospital, Kaohsiung, Taiwan, in a 12 hr light/dark cycle with lights on at 7 a.m. Pregnant rats were checked for litters daily at 10.0 h. Sprague-Dawley female rats were allowed to mate with male rats for 24 h. One day later, female rats were separated from the male rats and housed individually in a standard plastic home cage. After confirmation of pregnancy on the 14th day after mating, pregnant females were randomly divided for the prenatal steroid exposure paradigm or left undisturbed until delivery. The day of birth was designated as postnatal day 0 (PND 0). Rat pups were weaned at PND 21 and had access to standard chow and water* ad libitum*. Only male rats were used in this study.

### 2.2. Prenatal Dexamethasone Exposure Paradigm

Pregnant Sprague-Dawley rats at gestational days 14–21 were administered i.p. dexamethasone (0.1 mg/kg/day) [13]. Acute effects of prenatal programming by glucocorticoid were assessed in postnatal day 7 rats.

### 2.3. Melatonin Treatment

This may be the therapy drug for the pathway study. Since melatonin was used clinically in oral form, we added a new group with melatonin dissolved in bottle water. Rats drink about 25 mL/day and the average daily intake of melatonin was estimated to be 1 mg/kg/day from pregnant gestational days 14–21 to sacrifice. Melatonin was prepared three times a week by dissolving the melatonin (16 mg) in ethanol (1 mL, 100% v/v). This solution was then diluted with distilled water to a final concentration of 40 mg/L. The bottles were covered with aluminium foil to protect them from light.

### 2.4. Immunohistochemical Localization of Oil Red Stain and Analysis for the Liver Lipid Proteins Expression Study

We cut 2~3 *μ*m thick sections of the frozen liver tissue and mount them on coating slides. Tissue sections were incubated with 3% hydrogen peroxide for 10 minutes to block endogenous peroxidase activity. The sections were antigen retrieved in boiling citrate buffer by microwave for 12 min, stained with oil red O dissolved in 60% isopropanol for 15 min at room temperature, and rinsed in 60% isopropanol followed by washing in dH20. Tissue was counterstained in hematoxylin, washed thoroughly in dH20, and mounted in Aqua-Mount media (Thermo Fisher Scientific, Loughborough, UK). Secondary antibody was applied after extensive washing with PBS for 30 min at room temperature. Diaminobenzidine tetrahydrochloride was used as the substrate to detect antigen-antibody binding, and the sections were counterstained with Mayer's hematoxylin. The positive stained cells numbers were counted in the total five hundred hepatocytes in each group.

### 2.5. RNA Isolation and RT-PCR

The PCR was performed in DNA (10 ng) and mixed with 10 ul SYBR Green PCR Master Mix containing 10 n mol of forward and reverse primers, in a final volume of 20 ul. To quantitate the tissue amount of RNA, we used RT-PCR with the LightCycler 480 Real-Time PCR System (Roche Co., Germany). Total RNA was extracted from the liver tissue. For RT-PCR, the reagent mixture was prepared according to the protocol provided by the manufacturer (Protech Technology, Taipei, Taiwan). Two micrograms of total RNA were used to generate cDNA using an oligodeoxynucleotide primer (oligo dT15) following the protocol for transcription (Promega, Madison, WI). PCR was performed in 20 uL LightCycler 480 SYBR Green I Master (Roche Co., Germany) containing 10 n mol forward primers and reverse primers and approximately 10 ng cDNA. The primers sequences were as follows. The leptin primers were 5′-TCTCCGAGACCTCCTCCATCT-3′ as forward and 5′-TTCCAGGACGCCATCCAG-3′ as reverse. The *β*-actin was 5′-TCACCCACACTGTGCCCATCTACGA-3′ and 3′-GGTAACCGTTACTCGCCAAGGCGAC-5′, respectively. Amplification and detection were performed with the LightCycler 480 Real-Time PCR System with the following profile: 1 cycle of 95°C for 10 min and 40 cycles of 95°C for 15 s, 60°C for 30 s, and 72°C for 15 s. After amplification was completed, a final melting curve was performed with 2 min of denaturation at 95°C and then cooling to 60°C and heating slowly until 95°C (20 min) according to the dissociation protocol of the LightCycler 480 Real-Time PCR System. Real-time fluorescence measurement was read and a threshold cycle (CT) value for each sample was calculated by determining the point at which the fluorescence exceeded a threshold limit, that is, 10 times above the standard of the baseline. The CT value from the samples was plotted on the standard curve, and the copy number was calculated automatically. The validation experiments were done in triplicate and amplification efficiencies were validated.

### 2.6. Western Blotting Analysis

After specific treatment, tissues were dissected from samples and frozen immediately in liquid N_2_. The tissue was homogenized in a buffer and then centrifuged at 14,000 ×g. Protein (40 *μ*g) from the supernatant of each sample was separated by SDS-PAGE and transferred to polyvinylidene difluoride membranes by electrophoresis. The membranes were blocked in TBST buffer containing 5% low fat milk powder for 1 hr at room temperature. Immunoblotting assay was performed using a specific primary antibody: primary monoclonal mouse Histone 4, leptin, activated caspase 3 antibody, and tumor necrosis factor alpha (TNF-*α*) antibody followed by a secondary alkaline phosphatase-conjugated anti-IgG antibody (1 : 5000; Promega). The Western blots were visualized using the Blot AP System (Promega).

### 2.7. HDAC and DNMT Activity Assay

The measurement of histone deacetylases (HDAC) and DNA methyltransferase (DNMT) activity was performed using an EpiQuiktm HDAC and DNMT activity/inhibition assay kit (ET Epigentek, Farmingdale, NY) according to the manufacturer's instructions. For determination of HDAC and DNMT activity, 100 ug nuclear extracts from SD rat liver tissue at 6 ug nuclear protein concentrations were added to each strip well which contains stably captured antibody substrate. Samples were incubated at 37°C for 60 minutes to let each activity assay bind to an enzyme substrate. Subsequently, the high affinity acetylated histone antibody (1 mg/mL) was used to recognize undeacetylated substrate. The amount of the undeacetylated substrate was inversely proportional to enzyme activity. Finally, the enzymatic activities of HDAC and DNMT were detected using a microplate reader at 450 nm following an ELISA-like reaction. HDAC and DNMT activities were expressed as relative OD values per hour per mg of protein sample (OD/h/mg).

### 2.8. Bisulfite Modification

Bisulfite modification was performed based on the principle that bisulfite converts unmethylated cytosine residues into uracil, whereas methylated cytosine residues remain unaffected. Thus, after bisulfite conversion, methylated and unmethylated cytosines can be determined by different methods such as methylation-specific PCR (MSP) and direct sequencing. Bisulfite treatment of DNA was performed according to the manual instructions of Zymo methylation Gold kit (Zymo Company).

### 2.9. Methylation-Specific qPCR

The MSP was assessed as nested PCR with 2 ug of bisulfite-treated DNA in the first round of the PCR. The bisulfite-treated DNA 1 *μ*L was used for two separate nested PCR with 10 *μ*M of each primer specific for methylated and unmethylated sequences as follow.

The primers were reactions specific for bisulfite-converted DNA methylated or unmethylated sequences. The leptin methylated primers were 5′-GTTTAGTAGTTGTTGGTCGGATTTC-3 as forward and 5-CAACCTAATACTCCATTCTAAACGC-3 as reverse. The leptin unmethylated primers were 5-TTTAGTAGTTGTTGGTTGGATTTTG-3 and 5-AACCTAATACTCCATTCTAAACACC-3 as reverse. Amplification, detection, and after amplification were performed according to the protocol of the LightCyclerR 480 Real-Time PCR System.

### 2.10. TdT-Mediated dUTP-Biotin Nick End-Labeling (TUNEL)

We plan to study the cellular apoptosis expression in liver. The detection of TUNEL was according to the method of the protocol [14]. We used ApopTag Plus Peroxidase In Situ Apoptosis Detection Kit (CHEMICON International, Inc., USA) for TUNEL. Deparaffinized sections were washed with distilled water and treated with protein digestion enzyme for 15 min at 37°C. The positive stained cells numbers were counted in the total five hundred hepatocytes in each rat.

### 2.11. Immunohistochemistry

Formalin-fixed well-preserved SD rat tissue blocks from surgically resected liver specimens were used for immunohistochemical study. The 4 *μ*m sections of formalin-fixed tissues were mounted on silanized slides, deparaffinized in xylene, and rehydrated through serial baths of alcohol to water. The hydrated sections were treated in 3% hydrogen peroxide for 15 minutes to eliminate endogenous peroxidase activity and washed in phosphate-buffered saline (PBS).

The primary antibodies were used in this study for activated caspase 3 (1 : 250) mouse monoclonal antibody (cell signaling number 9661). The monoclonal antibody-treated slides were raised in PBS solution and incubated with a DaVinci Green (Biocare PD900, CA). The slides were washed in PBS and then incubated with MACH4 universal HRP polymer kit (Biocare M4U534, CA) for 15 minutes. After washing with PBS, a chromogenic reaction was developed by incubating with Betazoid DAB chromogen kit (Biocare BDB2004, CA).

### 2.12. Cytokine Secretion with Enzyme-Linked Immunosorbent Assay (ELISA)

The plasma was analyzed for the levels of cytokine using interleukin-6 (IL-6) and transforming growth factor-beta (TGF-*β*) commercial ELISA kits (R & D Systems, Minneapolis, MN) according to the manufacturer's protocols. A standard curve using recombinant cytokine was generated for each assay.

### 2.13. Statistical Analysis

SPSS for Windows 13.0 version was used for statistical analysis. Continuous variables were analyzed by independent *t*-test or ANOVA. The data was presented as means ± SE. *P* < 0.05 was considered to be statistically significant.

## 3. Results and Discussion

### 3.1. Liver Steatosis Study

**Figure 1:**
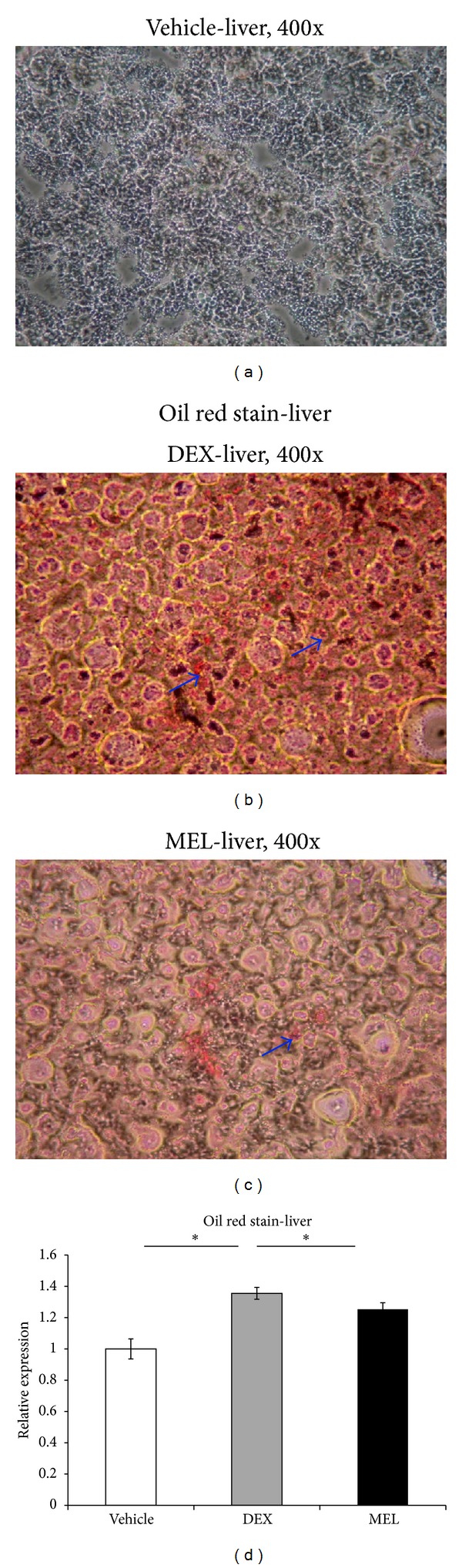
Oil red was overexpressed in prenatal steroid group more than in control group and downexpressed in melatonin group. (a) The oil red stain in the sham group, (b) the increased liver cell oil red stain in the prenatal steroid group (DEX), and (c) the melatonin decreasing the oil red stained cells induced by DEX at 7 days (original magnification ×400, arrows: positive hepatocytes). (d) Semiquantification of the oil red stained cells. All the results represent mean ± standard error of six animals; *P < 0.05 when comparing prenatal steroid groups. The letters above each represented different groups with DEX representing the prenatal steroid; MEL: melatonin treatment after prenatal steroid.

The liver steatosis was studied by oil red and it was overexpressed in the prenatal steroid group more than in the control group and downexpressed in the melatonin group (Figures 1(a)–1(d)).

### 3.2. The Expressions of Leptin and Methylation Study

**Figure 2:**
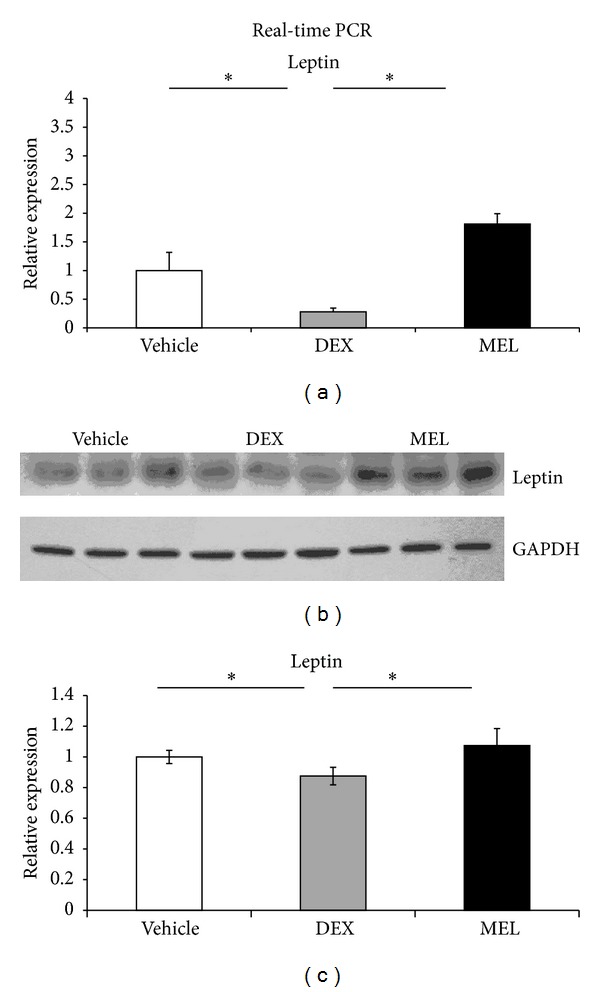
(a) In real-time PCR study, the leptin decreased in prenatal steroid group more than in control group and increased in melatonin group in liver. (b) Western blot showed decreased leptin expression in prenatal steroid group more than in control group and increased in melatonin group. (c) Semiquantification of the Western blot expression of leptin. All the results represent mean ± standard error of six animals; *P < 0.05 when comparing prenatal steroid groups with vehicle groups or melatonin groups with prenatal steroid groups. The letters above each represented different groups with DEX representing the prenatal steroid; MEL: melatonin treatment after prenatal steroid.

**Figure 3:**
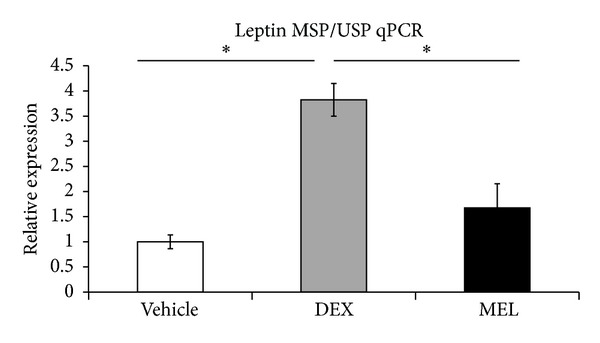
In leptin methylation-specific PCR/unmethylation-specific PCR (MSP/USP) study, the prenatal steroid groups increased the methylation and it decreased in melatonin group. All the results represent mean ± standard error of six animals; *P < 0.05 when comparing prenatal steroid groups with vehicle groups or melatonin groups with prenatal steroid groups. The letters above each represented different groups with DEX representing the prenatal steroid; MEL: melatonin treatment after prenatal steroid.

Real-time PCR showed that leptin decreased in prenatal steroid group more than in control group and recovered in melatonin group in liver (Figure 2(a)). Western blot showed decreased liver leptin expression in prenatal steroid group more than in control group and increased leptin in melatonin group (Figure 2(b)). The PEPCK and IGF1 protein expressions were not significantly different in the Western blot (data not shown). The total DNA methylation was studied by methylation-specific PCR/unmethylation-specific PCR (MSP/USP). It showed that the prenatal steroid groups increased the methylation and that it was decreased in melatonin group (Figure 3).

### 3.3. The Liver HDAC and DNMT Activity

**Figure 4:**
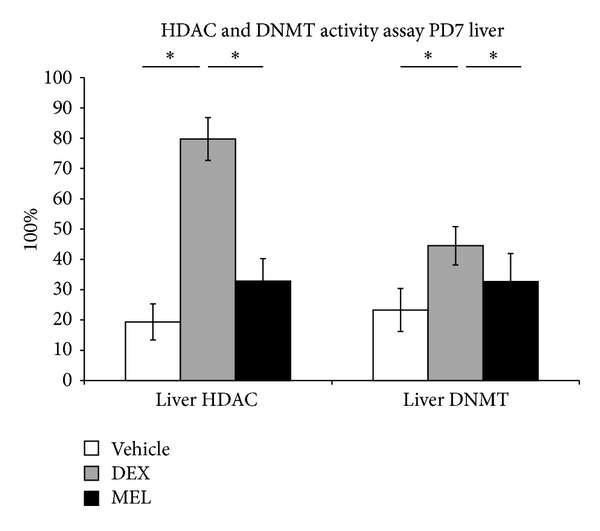
Liver HDAC and DNMT activity was increased in prenatal steroid group more than in control group and decreased in melatonin group. All the results represent mean ± standard error of six animals; *P < 0.05 when comparing prenatal steroid groups with vehicle groups or melatonin groups with prenatal steroid groups. The letters above each represented different groups with DEX representing the prenatal steroid; MEL: melatonin treatment after prenatal steroid.

The histone modifications in fatty liver were studied with the activity of HDAC and DNMT. Our data showed that the activity was increased in prenatal steroid group more than in control group and that it was decreased in melatonin group (Figure 4).

### 3.4. Apoptosis and Inflammation Study

**Figure 5:**
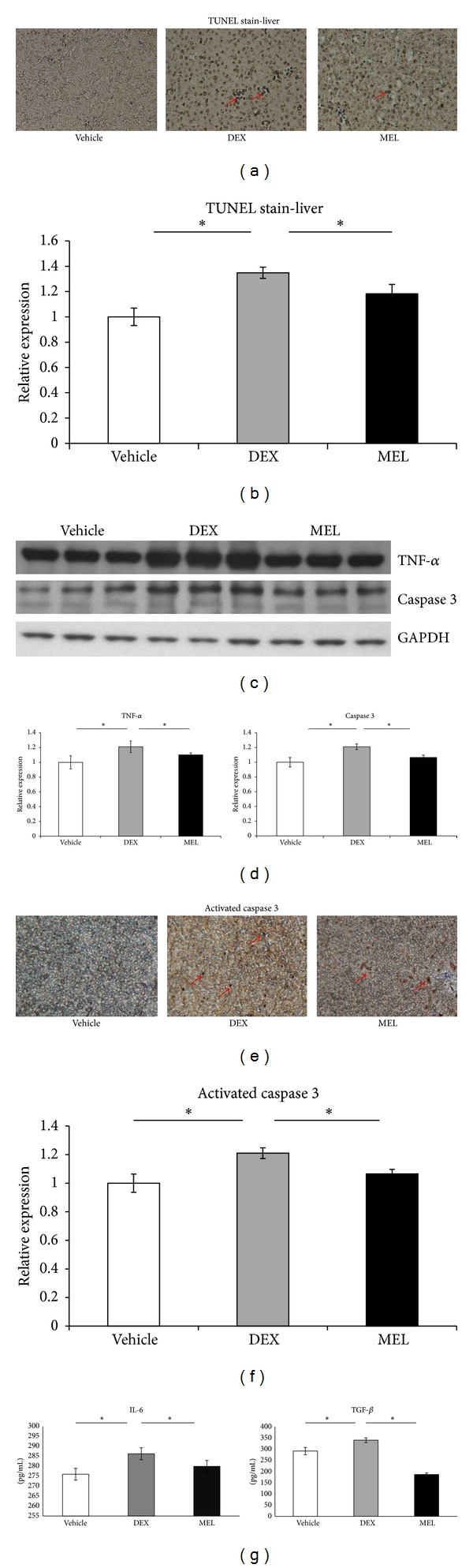
The apoptosis and inflammation study. The decreased apoptosis by TdT-mediated dUTP-biotin nick end-labeling (TUNEL) and activated caspase 3 expressions in melatonin group mildly increased in prenatal steroid group more than in control group. (a) The TUNEL stain showed the increased liver cell apoptosis in the prenatal steroid, and the melatonin decreased the apoptosis cells induced by prenatal steroid at 7 days (original magnification ×200, arrows: positive hepatocytes). (b) Semiquantification of the TUNEL stained apoptosis cells. (c) Western blot showed increased TNF-*α* and activated caspase 3 expression in prenatal steroid group more than in control group and decreased in melatonin group. (d) Semiquantification of the Western blots expressions of TNF-*α* and activated caspase 3. (e) The activated caspase 3 immunohistochemistry showed increased staining in the prenatal steroid group more than in control group and decreased in the melatonin group (original magnification ×200, arrows: positive hepatocytes). (f) Semiquantification of the activated caspase 3 immunohistochemistry staining. (g) ELISA study showed increased IL-6 and TGF-*β* levels in the prenatal steroid group more than in the control group and decreased in the melatonin group. *P < 0.05 when comparing prenatal steroid groups. All the results represent mean ± standard error of six animals. The letters above each represented different groups with DEX representing the prenatal steroid; MEL: melatonin treatment after prenatal steroid.

To assess whether apoptosis is involved in this liver damage, the activation of the apoptotic machinery was measured using the activated caspase 3 and the extent of TUNEL staining. Our data showed that apoptosis was mildly increased in prenatal steroid group than in control group and that it was decreased in the melatonin group (Figures 5(a)-5(b)). Activated caspase 3 and TNF-*α* proteins expression increased in prenatal steroid group more than in control group and it decreased in the melatonin group (Figures 5(c)-5(d)). Increased activated caspase 3 staining was noted in the prenatal steroid group more than in the control group and it decreased in the melatonin group (Figures 5(e)-5(f)). The IL-6 and TGF-*β* levels were increased in the prenatal steroid group more than in the control group and they decreased in the melatonin group (Figure 5(g)).

## 4. Discussion

Our data showed that melatonin rescued the prenatal glucocorticoid-induced liver steatosis and apoptosis in neonatal rats. One major mechanism was through histone activities which were activated by prenatal glucocorticoid and decreased after melatonin administration. The methylation mechanism of leptin was also involved in the regulation after melatonin administration which was activated by prenatal glucocorticoid.

Steatosis in nonalcoholic fatty liver disease (NAFLD) is the most common cause of chronic liver disease in US adults [15]. Prenatal glucocorticoid overexposure in rats increases hepatic lipid accumulation with steatosis [4]. Regulatory regions of the human genome can be modified through epigenetic processes during prenatal life to make an individual more likely to suffer from chronic diseases [4–6]. It was reported that this may be due to programmed alterations in fat metabolism [4]. In our study, this is also proved and this can be recovered by adding melatonin in the acute stage after delivery.

Leptin deficiency in mice and humans causes morbid obesity with fatty liver, and replacement leads to decreased food intake and increased energy expenditure [9, 16]. Low leptin levels can also be the result of rare genetic disorders such as lipodystrophies [9]. Some reported that leptin administration will correct many of the metabolic syndromes including hepatic steatosis [16, 17]. In our study, we showed that the decreased leptin expression in RT-PCR and Western blot in prenatal steroid group and the methylation of the leptin may be an important mechanism in this process.

Melatonin concentrations were elevated at night and lowered during the day [2], and chronic phase shifts on maternal and fetal hormonal and metabolic and circadian rhythms [2, 18]. Leptin concentrations were significantly affected by melatonin treatment [2, 19]. A recent report has demonstrated that the maternal melatonin secretion plays an important role in programming energy metabolism in the offspring [18]. In our study, we proved that the administration of melatonin in maternal gestation can reverse the leptin and leptin methylation in offspring. This gives our clinical new strategy to have melatonin treatment of prenatal stress with hepatic steatosis in fatty liver.

Are DNMT and HDAC associated with fatty liver disease and rescued by administration of melatonin? Distinctive genomic methylation patterns must be formed and maintained with high fidelity to ensure the inactivities of specific promoters during development [20]. Hepatic epigenetic phenotype predetermines individual susceptibility to hepatic steatosis in mice [5]. Histone deacetylation is catalyzed by HDAC, and the modification of core histones and transcription factors causes transcriptional activation [2]; while HDAC inhibitors exerted their antiadipogenic effect without inducing apoptosis or affecting cell viability and number [21]. In our study, melatonin decreased HDAC expression which was increased in prenatal steroid in the early stage. Loss of genomic and repetitive sequences cytosine methylation, especially at major and minor satellites, was accompanied by increased levels of repeat-associated transcripts, aberrant histone modifications, and alterations in expression of the maintenance DNMT proteins in the livers [5]. It is also found in our study that liver DNMT activity was increased in prenatal steroid and decreased after melatonin administration.

Apoptosis is the main process contributing to disease progression in NAFLD [22]. The significant association between steatosis and increased apoptosis suggests that hepatocyte lipid accumulation contributes to cell death [23]. It is reported that caspase 3 activation was markedly increased in the liver of patients with severe steatohepatitis and in that of those with simple steatosis [22, 24]. Hepatocyte apoptosis may directly or indirectly promote inflammation [25]. The response to noxious insults is typically deregulated, prolonged, and inflammatory in nature [26]. TNF-*α* can activate specific intracellular pathways in hepatocytes that influence cell proapoptotic signals via the caspases [27]; it is a critical inflammatory mediator involved in diseases [28]. A recent study has shown that Kupffer cell engulfment of apoptotic bodies results in the secretion of TNF-*α* along with increased expression of the death ligands like TNF-related apoptosis-inducing ligand (TRAIL) [29]. Since Kupffer cells are the major producers of the cytokines that modulate TNF-*α* and IL-6 activity, higher TNF-*α* expression in our prenatal steroid group suggests that steatosis was related to Kupffer cell dysfunction or activation [27, 30]. IL-6 acts as a proinflammatory cytokine [31]; it can induce hepatic inflammation and has an important role in the pathogenesis of fibrosis and diseases of the liver [32]. TGF-*β* signaling increased in fatty liver with inflammation [33–35] and TGF-*β* may induce apoptosis in numerous cell types [36]. In our study, the apoptosis is involved in this liver damage; the activation of the apoptotic machinery was measured with the higher expression of activated caspase 3 and the more extent of TUNEL staining in the prenatal steroid administration group. The inflammation may be involved in this process with higher IL-6, TNF-*α*, and TGF-*β* expression in this prenatal steroid administration.

## 5. Conclusions

In summary, our study showed that the prenatal glucocorticoid induced programming of liver steatosis in 7-day-old rat. The mechanism may be via reduced leptin expression with increased methylation. Melatonin can reverse the methylation, apoptosis, and the liver steatosis.

## Abbreviations

HDAC: Histone deacetylases
DNMT: DNA methyltransferase
IGF1: Insulin-like growth factor 1
IL-6: Interleukin-6
MSP/USP: Methylation-specific PCR/unmethylation-specific PCR
PEPCK: Phosphoenolpyruvate carboxykinase
PND: Postnatal day
TGF-*β*: Transforming growth factor-beta
TNF-*α*: Tumor necrosis factor-alpha
TUNEL: TdT-mediated dUTP-biotin nick end-labeling.
